# Supplementary material for: Genome Sequencing of the Japanese Eel (Anguilla japonica) for Comparative Genomic Studies on tbx4 and a tbx4 Gene Cluster in Teleost Fishes
Source: Mar Drugs. 2019 Jul 20;17(7):426. doi: 10.3390/md17070426 (PMC6669545; doi:10.3390/md17070426)
Supplement: Supplementary file 1 [file marinedrugs-17-00426-s001.zip › supplementary tables/Table S1.docx]

**Table S1.** Genome size estimation based on the 17-mer frequencies.

| **k-mer** | **K-mer number** | **Peak depth** | **Genome size (bp)** | **Used bases (bp)** | **Used**  **reads** | **Sequencing depth (×)** |
| --- | --- | --- | --- | --- | --- | --- |
| 17 | 37,982,773,125 | 37 | 1,026,561,436 | 44,121,403,125 | 383,664,375 | 43 |
