# Supplementary material for: Genome Sequencing of the Japanese Eel (Anguilla japonica) for Comparative Genomic Studies on tbx4 and a tbx4 Gene Cluster in Teleost Fishes
Source: Mar Drugs. 2019 Jul 20;17(7):426. doi: 10.3390/md17070426 (PMC6669545; doi:10.3390/md17070426)
Supplement: Supplementary file 1 [file marinedrugs-17-00426-s001.zip › supplementary tables/Table S2.docx]

**Table S2.** Summary of the genome sequencing data for the Japanese eel.

| **Insert size**  **(bp)** | **Sequencing length (bp)** | **Raw reads**  **(Gb)** | **Clean reads**  **(Gb)** |
| --- | --- | --- | --- |
| 270 | 150 | 80.92 | 71.66 |
| 500 | 120 | 27.27 | 24.51 |
| 800 | 120 | 21.72 | 19.63 |
| 2,000 | 120 | 29.77 | 20.89 |
| 5,000 | 120 | 33.52 | 17.69 |
| 10,000 | 120 | 33.72 | 15.19 |
| 20,000 | 120 | 41.67 | 14.48 |
| Total | --- | 268.61 | 184.05 |
