# Supplementary material for: Genome Sequencing of the Japanese Eel (Anguilla japonica) for Comparative Genomic Studies on tbx4 and a tbx4 Gene Cluster in Teleost Fishes
Source: Mar Drugs. 2019 Jul 20;17(7):426. doi: 10.3390/md17070426 (PMC6669545; doi:10.3390/md17070426)
Supplement: Supplementary file 1 [file marinedrugs-17-00426-s001.zip › supplementary tables/Table S3.docx]

**Table S3.** Statistics of the repeat sequences in the genome assembly of the Japanese eel.

| **Type** | **Repeat Size (bp)** | **Percentage of genome (%)** |
| --- | --- | --- |
| Trf | 74,462,905 | 6.57 |
| Repeatmasker | 85,570,095 | 7.55 |
| Proteinmask | 27,406,207 | 2.42 |
| De novo | 200,071,462 | 17.66 |
| Total | 259,849,065 | 22.94 |
