# Supplementary material for: Genome Sequencing of the Japanese Eel (Anguilla japonica) for Comparative Genomic Studies on tbx4 and a tbx4 Gene Cluster in Teleost Fishes
Source: Mar Drugs. 2019 Jul 20;17(7):426. doi: 10.3390/md17070426 (PMC6669545; doi:10.3390/md17070426)
Supplement: Supplementary file 1 [file marinedrugs-17-00426-s001.zip › supplementary tables/Table S4.docx]

**Table S4.** Statistics of the gene annotation for the assembled genome of the Japanese eel.

| **Gene number** | **Average gene length** | **Average CDS length** | **Average exon number** | **Average exon length** | **Average intron length** |
| --- | --- | --- | --- | --- | --- |
| 17,147 | 12,524 bp | 1,190 bp | 7.6 | 156 bp | 1,709 bp |

Abbreviation: CDS, coding sequence.
